# Chaotic Effect-Based Array Duffing Systems with Improved Nonlinear Restoring Force for Weak Signal Detection in Dynamic MWD

**DOI:** 10.3390/s23177598

**Published:** 2023-09-01

**Authors:** Yi Yang, Qian Ding, Yi Gao, Jia Chen

**Affiliations:** School of Electronic Engineering, Xi’an Shiyou University, Xi’an 710065, China

**Keywords:** array duffing system, frequency detection, parameters estimation, scale transformation, MWD

## Abstract

In the process of dynamic Measurement While Drilling (MWD), the strong vibration and rapid rotation of the Bottom Hole Assembly (BHA) lead to multi-frequency and high-amplitude noise interference in the attitude measurement signal. The weak original signal and extremely low signal-to-noise ratio (SNR) are always the technical difficulties of dynamic MWD. To solve this problem, this paper uses the chaotic effect of the Duffing system, which takes the expression (−*x*^3^ + *x*^5^) as a nonlinear restoring force to detect the weak characteristic signal of downhole dynamic MWD. First of all, in order to meet the limit condition of the chaotic phase transition of the system output, the frequency value of the characteristic signal is reconstructed and transformed based on the variable scale theory. Then, in order to solve the influence of the initial phase of the characteristic signal on the detection accuracy, a detection model based on the array Duffing system is presented, and a frequency-detection scheme with all-phase coverage is given. Finally, another array Duffing system is designed for the parameter estimation of the characteristic signal. The critical value of chaotic phase transition is determined by adjusting the amplitude of the driving signal of the array Duffing system, and then the amplitude and phase parameters of the characteristic signal are synchronously estimated. The experimental results show that the proposed method can effectively extract the weak characteristic signal within the strong noise, and the SNR of the characteristic signal can be as low as −21 dB. Through the attitude calculation for the extracted characteristic signal, it can be seen that the proposed method can improve the accuracy of the inclination of the drilling tool significantly, which proves the feasibility and effectiveness of the method proposed in this paper.

## 1. Introduction

The meaning of dynamic measurement while drilling (MWD) is to obtain real-time measurement data about the drill bit’s posture during the drilling process. Then, the obtained measurement data are transmitted to the ground through wireless transmission in real time, which provide reference opinions for the next steps of the staff operations. The acquisition of these measurement data is achieved by converting the analog measurement signals of various sensors in the measuring instrument into digital signals, and then calculating them based on physical models. Then, with the help of the drilling fluid (mud) inside the drill string, it is transmitted to the ground. Finally, the ground receives the mud pressure waveform through a pressure sensor, and decodes the waveform to obtain parameters.

During the signal transmission process, vibrations such as cutting the rock stratum, and the collision between the drill string and the borehole wall can cause strong mechanical vibrations in the drilling tool assembly. Therefore, the pressure wave of the mud pulse signal detected by the pressure sensor contains a large amount of noise signals, resulting in an extremely low signal-to-noise ratio (SNR) of the downhole attitude measurement signal [1,2]. The related research shows that the amplitude of the near-bit vibration signal is generally about 10 g, and the maximum can reach 30 g, while the amplitude of useful acceleration sensor signal is generally less than 1 g. Therefore, the SNR of the characteristic signal is usually as low as −20 dB, or even lower [3,4]. The weak acceleration signal is annihilated in the strong vibration background noise, which interferes with the accuracy of tool attitude measurement seriously, and even makes the dynamic MWD invalid [5,6,7]. Therefore, how to detect weak useful signals from strong noise backgrounds with unfixed frequencies has become a major issue in mud pulse-transmission technology.

The digital filter based on time–frequency analysis was first applied to the noise suppression of MWD signals. Tu [8] develops a set of ground decoding systems and uses finite impulse response (FIR) digital filtering to make noise reduction on the mud pulse signal. The field experiment results show that the developed device can correctly extract and recognize the mud pulse signal with a simple and practical decoding process. Zhao [9] uses a combination of linear filtering and a nonlinear “flat-top elimination” method to process the signals collected at the drilling field. The comprehensive comparison with the processing results of the linear filtering method shows that this method can more effectively remove the noise and other interference of drilling pump.

Digital filtering is a signal processing method based on noise suppression. Its realization is based on the premise that the spectrum of the characteristic signal does not overlap with the spectrum of noise interference. Under such conditions, it can retain useful signals and filter out irrelevant noise components during the filtering process [10]. However, in the actual collected MWD signals, the frequency distribution of noise signals is very complex due to the variety of interference sources, and there is bound to be a part close to the frequency of the characteristic signal [11,12]. Therefore, while suppressing noise, the characteristic signal will inevitably be suppressed or damaged, and even lead to invalid MWD.

In order to improve the accuracy of attitude measurement for the drilling tool, some scholars refer to the attitude measurement principle in the field of integrated navigation. The advanced filtering algorithm is applied to the downhole sensor signal to eliminate the noise interference maximum and realize its optimal estimation. Ref. [13] proposed a linear random model by using a three-axis accelerometer and a three-axis magnetometer to form an MWD system, and then the state of the model is estimated by the Kalman Filter (KF) algorithm. However, the drilling tool is affected by many kinds of vibration during the drilling process, and shows strong nonlinear characteristics. Therefore, there is an obvious system model error between the linear stochastic system and the real drilling dynamic system, while the KF algorithm is only applicable to linear systems, and it cannot be used for iterative calculation of multi-sensor nonlinear systems.

Aiming at the above problems, refs. [14,15,16] established a nonlinear model of downhole attitude measurement based on quaternion, and used the unscented Kalman Filter (UKF) and its improved algorithm to filter the vibration interference signal. The simulation results prove the effectiveness of this method. However, the application of UKF needs to meet the assumptions that the system state model and measurement model are accurately known. For the actual measured system in the drilling process, it is difficult to accurately establish an appropriate system model due to the influence of the complex underground environment. The predetermined system model has uncertainties such as system parameter mutation, instantaneous interference, unknown statistics of system noise, unknown drift, etc., which leads to the decline of the estimation accuracy of the filter. Even filtering divergence occurs, which makes the filter lose its estimation function [17]. Therefore, the existing dynamic measurement methods based on multi-sensor combined measurement and optimal estimation algorithms have defects and limitations.

With the rapid development of science and technology, some emerging signal processing methods have been applied to weak signal detection in dynamic MWD [18,19,20]. Ref. [21] proposes a method of downhole mud pulse signal recognition based on deep learning. The detection model is composed of a wavelet neural network and automatic encoder, which can achieve a good recognition effect. However, this method has the disadvantages of a difficult selection of wavelet parameters and dependence on subjectivity. Moreover, the SNR of the signal that can be processed is high, and the engineering applicability needs to be further verified. Ref. [22] adopts the set empirical mode decomposition (EEMD) method to establish the noise reduction and shaping algorithm of pulse signals. Then, the judgment criteria of the excellent noise reduction algorithm were presented for pulse signals while drilling was based on the algorithm approximation index and correlation index. The simulation results show that the proposed method is reasonable and effective. However, when the method is applied, it needs to reconstruct the signal one by one to calculate the mean square error value, making the algorithm less efficient. Moreover, the algorithm is unstable when building different low-pass filtering algorithms based on the natural mode.

In recent years, the profound study for nonlinear science has provided a new way for the researcher to understand, analyze and solve the problem of weak signal detection. The discovery of nonlinear dynamic phenomena such as chaos, fractal and stochastic resonance poses a powerful challenge to traditional signal processing methods [23,24,25,26]. Applying chaos theory to signal detection is a new research field rising from the 1990s. Compared with the traditional weak signal detection method, the weak signal detection method based on the chaotic phase transition can detect a lower SNR of the signal, and is not limited by the statistical characteristics of noise [27,28].

As a typical nonlinear model that can produce chaotic effects, the second-order Duffing system can realize the transformation of the output state under the conditions of appropriate parameters [29,30]. When the Duffing system is in the critical state from chaotic to large-scale periodic, it has the outstanding features of being sensitive to weak periodic signals and immune to noise signals. In addition, the greater the noise intensity, the more stable the chaotic state, and the higher the accuracy of weak signal detection through system phase transition [31,32,33]. This abnormal effect and advantage make the Duffing system important in the practical application of weak signal detection. Therefore, the application of chaos theory to the detection of weak signals while drilling has great research space and application value.

In conclusion, this paper presents a weak signal detection method for dynamic MWD based on the chaotic effect of the array Duffing system. Using the transition of the output state of the array Duffing system, the frequency detection and parameter estimation of the downhole attitude measurement signal are carried out, and then the complete sensor signal is obtained. Finally, real-time and accurate measurement of attitude parameters for drill tool is realized.

## 2. Frequency Detection

### 2.1. Basic Principle of Duffing Chaos Detection

The use of chaotic systems to detect weak signals was first proposed between 1990 and 1992. Based on the sensitivity of chaotic systems to initial conditions, the weak signal is taken as the initial condition of the chaotic system, and the chaotic system whose dynamic behavior is extremely sensitive to the weak signal is established. Putting the chaotic system in a specific state introduces the weak signal into this system, and then the weak signal covered by noise based on changes in the phase trajectory of the chaotic system is detected. This is the basic principle of using chaotic systems to detect weak signals. Therefore, establishing a chaotic system sensitive to weak signals is the primary condition for signal detection.

Chaos is a unique solution of some nonlinear equations. In other words, it is a unique motion form of some nonlinear systems. Chaotic motion has randomness, but the equations describing its motion are deterministic, such as the famous Duffing equation, Lorenz equation, etc. This article takes the Duffing equation as the research object to detect the dynamic MWD signals. The typical Duffing equation is as follows.

*x*” + *kx*’ − *x + x*^3^ *= λ*cos(*ωt*) (1)

In Equation (1), *x* is the solution of the equation, also known as the system output. *k* is the damping coefficient. According to the experimental results in ref. [34], parameter *k* is set as 0.5 in this article. The expression (−*x* + *x*^3^) is the nonlinear restoring force. *λ*cos(*ωt*) is a periodic driving force, that is, a driving signal, where *λ* and *ω* are, respectively, the amplitude and angular frequency of the driving signal. Moreover, the angular frequency *ω* is equal to 1 rad/s and *t* represents the time of the driving signal.

The Duffing equation is a motion equation, which describes a nonlinear elastic system. When the external signal is determined, the system performance mainly depends on the nonlinear restoring force of the system. Researches have shown that when the nonlinear restoring force is (−*x + x*^3^), the Duffing equation is not very sensitive to the response of sinusoidal signals [34]. However, the dynamic MWD signals to be measured in this article are weak sinusoidal signals. Therefore, the sensitivity of the Duffing equation to weak sinusoidal signals can be improved by increasing the power of the nonlinear restoring force terms. However, it is not the case that the higher the power of the nonlinear restoring force, the stronger the sensitivity of the Duffing system to sinusoidal signals. When the power of the nonlinear restoring force is enhanced further, it can be seen that the performance improvement of the Duffing system is not significant, and the higher power poses a significant obstacle to the calculation of the system’s chaos criterion [34]. Therefore, this article selects (−*x*^3^ *+ x*^5^) as the nonlinear restoring force. 

Based on the above analysis, the expression of the improved Duffing equation is as follows.

*x*” + *kx*’ − *x*^3^ *+ x*^5^ *= λ*cos(*ωt*) (2)

where the meaning of all parameters is the same as Equation (1).

Due to the existence of the nonlinear restoring force term, Equation (2) has rich nonlinear dynamic characteristics. The specific performance is that the output state of the system changes regularly with the increase in the amplitude *λ*, and successively goes through homoclinic orbit, chaos and a large-scale periodic state.

Small changes in the amplitude of the driving signal can cause significant changes in the system state. Therefore, set the amplitude as the critical value of the transition from chaotic state to large-scale periodic state or a value slightly less than the critical value, and add the noise signal and the characteristic signal, which has the same frequency as the driving signal in Equation (2). Finally, the weak signal detection model based on the Duffing system is as follows.

*x*” + 0.5*x*’ − *x*^3^ *+ x*^5^ *= λ_c_*cos(*t*) + *a*(*t*) + *n*(*t*) (3)

where *λ*_c_ is a little less than the critical value of the transition from the chaotic state to the large-scale periodic state, which is about 0.72 through a large number of experimental verifications. *a*(*t*) is the characteristic signal. If *a*(*t*) = 0.01cos*t*, it means a weak periodic signal with the same frequency is the driving signal. *n*(*t*) is the noise signal. If its strength *D* = 0.016, then *n*(*t*) = (2 × 0.016)^1/2^ × *g*(*t*), where *g*(*t*) is a Gaussian noise signal with a mean value of 0 and a variance of 1.

**Figure 1:**
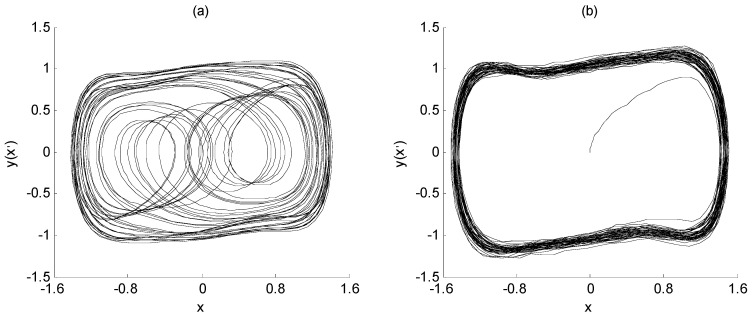
The output state of Duffing chaos system: (**a**) chaotic state; (**b**) large-scale periodic state.

Define SNR = 10 × log(*p*_a_/*p*_n_), where *p*_a_ represents the mean square value of characteristic signal *a*(*t*), and *p*_n_ represents the strength of interference signal *n*(*t*). From this, the SNR of the characteristic signal and the noise interference signal in Equation (3) is about −25 dB. At this time, it is difficult to extract the characteristic signal directly from the noise *n*(*t*) through spectrum analysis. However, if the Duffing chaos system is placed in the critical state of the transformation from a chaotic state to a large-scale periodic state, and the signal *a*(*t*) + *n*(*t*) is taken as the input of the Duffing chaos system in this state, the frequency value of the characteristic signal can be identified from the output state of the Duffing chaos system, which is Equation (3). When the signal *a*(*t*) + *n*(*t*) is not considered, the output state of the Duffing chaos system is shown in Figure 1a. On the other hand, while the noisy signal *a*(*t*) + *n*(*t*) is added, the output state of Duffing chaos system is shown in Figure 1b. The ordinate *y* in the figure represents *x*’.

The X-axis of Figure 1 represents the solution of the Duffing equation, which is the system output response x. The Y-axis of Figure 1 represents the first derivation of the system output response, which is x’. They are all dimensionless quantities. If plotting these two quantities on a plane, the phase diagram of the Duffing system is achieved as shown in Figure 1. During the simulation, both the initial values of x and x’ are set to 0, that is, x(0) = 0; x’(0) = 0. Therefore, the starting points of the phase trajectories of both images are at the zero point of the two-dimensional coordinate axis.

From the changes in Figure 1a,b, since the angular frequency of the characteristic signal is the same as that of the system drive signal, the input of the characteristic signal can make the amplitude of the drive signal exceed the critical value, and further make the system change from a chaotic state to a large-scale periodic state. In addition, although the characteristic signal contains noise interference to a certain extent, it does not affect the change in the system state, and only makes the large-scale periodic state rough. Therefore, the weak signal detection method based on the Duffing system has immunity to noise. This is the basic principle of frequency detection for the weak signal using the chaotic effect of the Duffing system.

Although the chaotic effect of the Duffing system can successfully identify weak signals under a strong noise background, the following problems still exist when it is applied to the detection of weak characteristic signals in dynamic MWD.

(1) According to the basic principle of Duffing system weak signal detection, the premise of frequency detection through Equation (3) is that the frequency value of the driving signal is consistent with that of the characteristic signal. So, the angular frequency of the characteristic signal is required to be known. However, due to the combined effects of drilling tool vibration, cutting and the movement of drill string, the angular frequency of dynamic MWD signal is not a constant value, but fluctuates constantly based on the rotation speed of drill string, which makes the application of the Duffing system more difficult.
(2) While keeping other parameters of the Duffing system unchanged, the change in driving signal frequency will affect the critical amplitude of the system phase transition. This means that when the frequency of the MWD signal is constantly changing, it is necessary to repeatedly calculate the critical amplitude of the system at this frequency value. Obviously, the amount of calculation increases sharply in this case, which cannot meet the real-time requirements of dynamic MWD.
(3) In the Duffing system, if the angular frequency of the driving signal exceeds 1 rad/s, the dynamic response characteristics of the system will deteriorate, and the chaos and large-scale periodic states do not easily appear [28]. However, the angular frequency of the characteristic signal is mainly determined by the rotation speed of the drill string in MWD, and changes between 2π and 6π during normal drilling. It obviously exceeds the limit of Duffing system chaos detection on the frequency value, and will seriously affect the feasibility and accuracy of detection.

### 2.2. Frequency Detection Based on Array Duffing Chaos System

#### 2.2.1. Large Frequency Detection Method Based on Variable Scale Duffing System

Aiming at the problems of the Duffing system while it is applied to dynamic MWD, this section adjusts the Duffing system shown in Equation (3) based on scale transformation theory, and establishes a chaos detection scheme for large frequency parameters. The key to this problem is how to reconstruct and transform the frequency value of MWD signals (characteristic signals).

Assume the characteristic signal *a*(*t*) = *γ*cos(*ωt*), where *t* represents the time of the characteristic signal. Keep the other system parameters in Equation (3) unchanged, and introduce a transformation coefficient *N*. So, the characteristic signal is magnified *N* times on the time axis, that is, *t*_1_ = *N*·*t*. For MWD signals, *N* ∈ [2π, 6π]. Assume the angular frequency of the characteristic signal *ω* = *N* ≠ 1 rad/s, then the characteristic signal is updated to the following form.

*a*(*t*_1_) = *γ*cos(*ω*·*t*) = *γ*cos(*N*·*t*_1_/*N*) = *γ*cos(*t*_1_) (4)

It can be seen from Equation (4) that the angular frequency of the MWD signal is compressed from *N* to 1 rad/s on the frequency axis. At this time, it is possible to detect whether the signal *s*(*t*_1_) exists under the action of Equation (3), and then determine the frequency value of the characteristic signal according to the relationship of *ω* = *N*. However, it is impossible to directly reconstruct the signals collected by downhole sensors through the linear transformation shown in Equation (4) when processing actual measured signals. Therefore, the scale transformation of the numerical calculation step is considered to realize the reconstruction of the frequency value. The specific steps are as follows.

(1) Assuming the sampling frequency is *f*_s_, the step size of the numerical calculation should be as follows.

*T* = 1/*f*s (5)

(2) The transformation coefficient R is introduced, and the step size of numerical calculation is updated to the following form.

*T*_1_ = *N*·*T* = *R*/*f*s (6)

At this time, the time interval of the signal is increased by *N* times, and the corresponding signal angular frequency is compressed *N* times. In other words, an MWD signal with a sampling frequency of *f*_s_ and an angular frequency of *ω* is transformed into a signal with a sampling frequency of *f*_s_/*N* and an angular frequency of *ω*/*N* through variable scale processing.

(3) Input the above signals into the Duffing detection system and make the calculation step meet Equation (6) to complete the parameter reconstruction of the MWD signals, and then the frequency value of the characteristic signal is identified by the transition of the output state of the Duffing system.

From the above analysis, it can be seen that scale transformation does not change the value of the characteristic signal, but only changes the frequency (or time) scale of the signal, compressing (or enlarging) it on the time axis and reordering the values, which will not affect the output result of the Duffing system.

According to the above principle of the variable scale Duffing system for frequency detection, no matter how the frequency value of the characteristic signal changes, it is reduced to 1 rad/s. In other words, the frequency of the characteristic signal is consistent with the angular frequency of the driving signal through scale transformation processing. Therefore, a fixed set of system parameters is always used in the detection process. At this time, the determination of the critical amplitude of the system is independent of the frequency value of the characteristic signal. Thus, problems (1) and (2) proposed in Section 2.1 have been solved successfully. The frequency of the MWD signal is compressed by the transforming coefficient to make it meet the detection conditions of the Duffing system. As a result, problem (3) proposed in Section 2.1 has also been solved.

#### 2.2.2. All-Phase Frequency Detection Based on Array Duffing Chaos System

**Figure 2:**
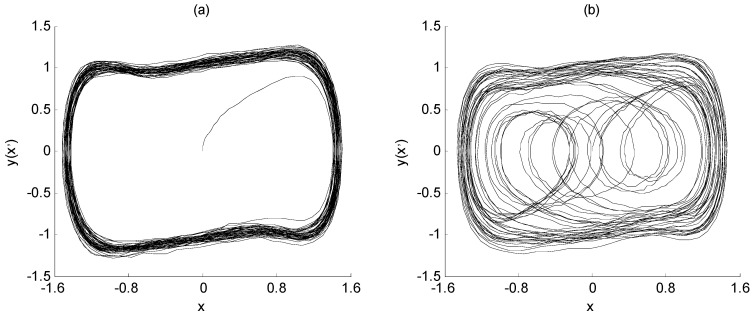
The output state of Duffing chaos system: (**a**) while the initial phase angle of the characteristic signal is 0; (**b**) while the initial phase angle of the characteristic signal is π.

Based on the analysis above, it is always assumed that the initial phase angle of the characteristic signal and the driving signal is 0. However, for the actual MWD signal, the initial phase is not always equal to 0. Therefore, it is necessary to study the influence of the initial phase of the characteristic signal on the frequency detection of the Duffing system. In Equation (3), take the characteristic signal *a*(*t*) as 0.01cos*t* and 0.01cos(*t* + *π*), respectively, and other parameters remain unchanged. The output state of the Duffing chaos system are shown in Figure 2.

As can be seen from Figure 2, when the initial phase angle of the characteristic signal is not 0, even if its frequency is consistent with the driving signal, the output state of the Duffing system is still chaotic. Then, the characteristic signal cannot be detected through the phase state transition. It can be seen that the initial phase angle of the characteristic signal will indeed affect the effectiveness of Duffing system detection.

To solve this problem, this section establishes an all-phase frequency detection scheme based on the array Duffing system. Because the angular frequency of the characteristic signal will eventually be adjusted to 1 rad/s by scale transformation, in order to simplify the analysis, the angular frequency of the characteristic signal is directly set to 1 in this section. In addition, the amplitude of the driving signal is still set to 0.72. Therefore, the Duffing system frequency detection model after considering the initial phase angle is as follows.

*x*” + 0.5*x*’ − *x*^3^ *+ x*^5^ *=* 0.72cos(*t + β*) + *γ*cos(*t + θ*) + *n*(*t*) (7)

where *γ* is still the amplitude of the characteristic signal, *β* and *θ* represent the initial phase angle of the driving signal and the characteristic signal, respectively. The value range of *θ* is from −π to π.

If the first two terms at the right end of Equation (7) are simplified, the following form can be obtained.

(8) $$\begin{array}{l} 0.72\cos\left(t+\beta \right)+\gamma \cos\left(t+\theta \right)=\\ \left(0.72\cos t\cos\beta -0.72\sin t\sin\beta )+(\gamma \cos t\cos\theta -\gamma \sin t\sin\theta \right)\\ =(0.72\cos\beta +\gamma \cos\theta )\cos t-(0.72\sin\beta +\gamma \sin\theta )\sin t \end{array}$$

Assume that *A* and *B* are the two right sides of a right triangle, and *C* is the oblique side. *A*, *B* and *C* are represented by the following equations.

(9) $$\left\{ \begin{array}{l} A=0.72\cos\beta +\gamma \cos\theta \\ B=0.72\sin\beta +\gamma \sin\theta \\ C=\sqrt{{\left(0.72\cos\beta +\gamma \cos\theta \right)}^{2}+{\left(0.72\sin\beta +\gamma \sin\theta \right)}^{2}} \end{array}\right.$$

If sin*δ* = *B*/*C*, cos*δ* = *A*/*C*, then Equation (7) can be further simplified as follows.

(10) $$\begin{array}{ll} & 0.72\cos(t+\beta )+\gamma \cos(t+\theta )\\ = & A\cdot \cos t-B\cdot \sin t=C\cdot \cos\delta \cdot \cos t-C\cdot \sin\delta \cdot \sin t=C\cdot \cos(t+\delta )\\ = & \sqrt{0.72^{2}+1.44\gamma \cos\beta \cos\theta +1.44\gamma \sin\beta \sin\theta +\gamma^{2}}\cdot \cos(t+\delta )\\ = & \sqrt{0.72^{2}+1.44\gamma \cos(\theta -\beta )+\gamma^{2}}\cdot \cos(t+\delta ) \end{array}$$

It can be seen from Equation (10) that the characteristic signal and the driving signal are combined into a trigonometric function term. According to the position of *δ* in Equation (10), the value of *δ* has little effect on the amplitude parameters of the Duffing system, only having an effect on the initial position of the trajectory solution. What really affects the detection result is the amplitude term of the trigonometric function in Equation (10). Therefore, the expression of the amplitude term is established as follows.

(11) $$\sqrt{0.72^{2}+1.44\gamma \cos(\theta -\beta )+\gamma^{2}}\geq 0.73$$

The right end of Equation (11) is the minimum amplitude that can cause the output state of the Duffing system to change. Therefore, when Equation (11) holds, the total amplitude of the input signal will exceed the critical value, and the output state of the system will change from chaos to large-scale period. Further, the characteristic signal is identified by the change in phase state. If the amplitude *γ* of the characteristic signal is known, the identifiable initial phase range of the characteristic signal can be solved according to Equation (11). The amplitude of the MWD signal is usually greater than 0.1, and the minimum is not less than 0.02. Therefore, the amplitude of the characteristic signal is set to 0.02 and substituted into Equation (10). The following relationship can be obtained.

−arccos[(0.73^2^ − 0.72^2^ − 0.02^2^)/(1.44 × 0.02)] ≤ *θ* − *β* ≤ arccos[(0.73^2^ − 0.72^2^ − 0.02^2^)/(1.44 × 0.02)] (12)

By solving Equation (12), the variation range of term *θ*-*β* is from −60.7° to 60.7°. The simulation results also prove that when *β* = 0 and the other conditions remain unchanged, the variation range of *θ* is from −60° to 60°. Only when *θ* is within this range can Equation (7) realize the change in output state of the Duffing system. It is basically consistent with theoretical calculation. In addition, when the amplitude of the characteristic signal is greater than 0.02, the variation range of *θ* increases correspondingly according to the calculation results of Equation (12). Therefore, the above variation range of *θ* is applicable to all characteristic signals with an amplitude not less than 0.02.

**Figure 3:**
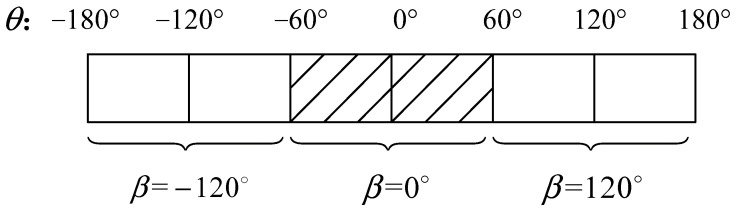
The detectable window of array Duffing chaos system.

From the above analysis, when other parameters of the system are determined, the initial phase angle of the characteristic signal must be within a certain range to effectively obtain the frequency of the characteristic signal, that is, there is a detectable window for the initial phase angle of the characteristic signal. The detection window is shown in Figure 3, in which the shaded part indicates the detectable window for *θ* while *β* = 0.

It can be seen from Figure 3 that if the detection window is to be fully covered in the whole range [−π, π], it can be realized by adjusting the initial phase angle of the drive signal. Specifically, when the initial phase angle *β* of the drive signal is set as −120°, the corresponding detection window of *θ* is from −180° to −60°. When *β* is set as 120°, the corresponding detection window of *θ* is from 60° to −180°. Therefore, in this section, the tradition Duffing system, which was shown in Equation (7), will be expanded to an array Duffing system which is composed of three Duffing equations with different initial phase angles of driving signals. Then, the all-phase frequency detection of characteristic signals is realized. The array Duffing system for all-phase frequency detection is as follows.

(13) $$\left\{ \begin{array}{l} x^{\prime \prime }+0.5x^{\prime }-x^{3}+x^{5}=0.72\cos(t+0^{\circ })+\gamma \cos(t+\theta )+n(t)\\ x^{\prime \prime }+0.5x^{\prime }-x^{3}+x^{5}=0.72\cos(t-120^{\circ })+\gamma \cos(t+\theta )+n(t)\\ x^{\prime \prime }+0.5x^{\prime }-x^{3}+x^{5}=0.72\cos(t+120^{\circ })+\gamma \cos(t+\theta )+n(t) \end{array}\right.$$

When the amplitude of the characteristic signal is greater than 0.02, it is substituted into the array Duffing equations, respectively. Because the initial phase of the signal detected by these three equations covers the whole range. Therefore, as long as the output state of one of the equations changes, the input signal can be identified as a weak characteristic signal with the same frequency as the drive signal, and then the frequency value can be determined. It can be seen that the influence of the initial phase angle of the characteristic signal on its frequency detection can be effectively solved through the array Duffing system.

It should be pointed out that according to the Lyapunov exponent analysis method, when the initial phase angle of the driving signal *β* is equal to 0, the effect of the system output phase transition can also be achieved. At this point, it is necessary to further increase the amplitude of the driving signal. Taking this article as an example, when the sum of the amplitudes of the driving signal and the signal to be measured is greater than 0.75 and the initial phase angle of the driving signal *β* is equal to 0, regardless of the initial phase angle of the signal to be measured, the frequency value of the signal to be measured can be detected through the system output phase change. However, in application, this method requires continuous adjustment of the amplitude of the driving signal to ensure that the amplitude of the two signals added is greater than 0.75. Therefore, the calculation amount of this method is relatively large and the calculation results are influenced by the amplitude of the signal to be measured. Based on the above analysis, the proposed all-phase frequency detection based on the array Duffing Chaos system in this article is a more suitable method for the dynamic MWD signal.

## 3. Parameter Estimation

It can be seen from the above analysis that the frequency value of the MWD signal in the strong noise background can be identified using the array Duffing equations combined with the scale transformation. However, in order to calculate the real-time attitude information of the drilling tool, it is also necessary to determine the amplitude and phase value of the characteristic signal, so as to recover the complete signal waveform. To solve this problem, an amplitude and phase synchronization estimation method based on the array Duffing system will be presented in this section.

According to the analysis results in the previous section, when Equation (11) holds, the output state of the Duffing system will change from chaotic to large-scale periodic. Assuming that λ_1_ represents the driving signal amplitude when the output state of the Duffing system just changes from chaotic to large-scale periodic, and then Equation (11) can be rewritten as follows.

(14) $$\sqrt{\lambda_{1}^{2}+2\lambda_{1}\gamma \cos(\theta -\beta )+\gamma^{2}}=0.73$$

where the *λ*_1_ could be determined by observing the output status of the Duffing chaos system.

After *λ*_1_ is determined, Equation (14) is a binary equation with an amplitude *γ* and phase *θ* of characteristic signals as variables. Therefore, another binary equation about amplitude and phase could be established in a similar way, and then the amplitude and phase values of the characteristic signal can be obtained by solving the binary function equations. It should be noted that since the variation range of *θ* is from −π to π, the absolute value of *θ* is solved through the binary function equation instead of *θ*. To further determine the value of *θ*, one more equation needs to be added. Therefore, aiming at the problem of parameter estimation to be solved in this section, a chaotic detection model based on array Duffing system is established as follows.

(15) $$\left\{ \begin{array}{l} x^{\prime \prime }+0.5x^{\prime }-x^{3}+x^{5}=\lambda_{1}\cos(t+0^{\circ })+\gamma \cos(t+\theta )+n(t)\\ x^{\prime \prime }+0.5x^{\prime }-x^{3}+x^{5}=\lambda_{2}\cos(t+180^{\circ })+\gamma \cos(t+\theta )+n(t)\\ x^{\prime \prime }+0.5x^{\prime }-x^{3}+x^{5}=\lambda_{3}\cos(t+90^{\circ })+\gamma \cos(t+\theta )+n(t) \end{array}\right.$$

Firstly, the initial phase of the drive signal is set to 0, 0.5π and π, as shown in Equation (15). Then, the characteristic signal is input into the array Duffing equations in Equation (15), respectively, and the amplitude of the driving signal is adjusted step by step. Finally, the output state of the Duffing system is observed and the amplitude of the system is recorded when the phase transition occurs.

When the output state of the array Duffing system changes from the chaos to large-scale period, its driving signal amplitude is expressed as *λ*_1_, *λ*_2_ and *λ*_3_. Therefore, the array Duffing equations for the amplitude *γ* and phase *θ* of the characteristic signal can be expressed as follows.

(16) $$\{\begin{array}{c} \begin{array}{l} \lambda_{1}^{2}+2\lambda_{1}\gamma \cos\theta +\gamma^{2}=0.73^{2}\\ \lambda_{2}^{2}-2\lambda_{2}\gamma \cos\theta +\gamma^{2}=0.73^{2} \end{array}\\ \lambda_{3}^{2}+2\lambda_{3}\gamma \sin\theta +\gamma^{2}=0.73^{2} \end{array}$$

The following results can be obtained by solving the above equation.

(17) $$\{\begin{array}{c} \gamma =\sqrt{0.73^{2}-\lambda_{1}\lambda_{2}}\\ \theta =\arccos\left(\frac{\lambda_{2}-\lambda_{1}}{2\sqrt{0.73^{2}-\lambda_{1}\lambda_{2}}}\right)\cap \arcsin\left(\frac{\lambda_{1}\lambda_{2}-\lambda_{3}^{2}}{2\lambda_{3}\sqrt{0.73^{2}-\lambda_{1}\lambda_{2}}}\right) \end{array}$$

The equations above is the estimation formula for the amplitude and phase of the characteristic signal. It can be seen that the array Duffing system used for parameter estimation in this section and the array Duffing system used for frequency detection in the previous section are derived based on Equation (10).

But the difference is that in Section 2.2.2, in order to analyze the influence of the initial phase of the characteristic signal on the frequency detection, the range of the initial phase value is determined by giving the amplitude of the driving signal and the characteristic signal, and then the all-phase array Duffing system for frequency detection is obtained. However, the model of the array Duffing system is first given in this section and the amplitude of its driving signals are determined by observing the output state of the array system. Finally, the amplitude and phase values of the characteristic signal are determined by solving Equation (16).

Based on the analysis above, the specific process of the chaotic effect-based improved array Duffing systems for dynamic MWD signal detection is as follows.

Step 1 Determine the parameters of Duffing system. For example, in Equation (3), the damping ratio *k* is set to 0.5, the angular frequency of the drive signal is set to 1 rad/s, the initial value of the system (*x*(0), *x*’(0)) is set to (0,0), and the amplitude of the drive signal *λ* is set to 0.72.

Step 2 Input the noise characteristic signal into Equation (7), and set the initial phase angle of the driving signal to be 0, ±2π/3 (the amplitude of the characteristic signal is required to be not less than 0.02), from which the array Duffing system for frequency detection is obtained.

Step 3 By introducing the transformation coefficient *R*, the characteristic signal with sampling frequency of *f*_s_ and angular frequency of *ω* is updated to the signal with sampling frequency of *f*_s_/*R* and angular frequency of *ω*/*R*. Then, the array Duffing equation for frequency detection is solved with the calculation step *T*_1_ = *R*/*f*_s_, and the phase trajectory of the output state of the array Duffing system is obtained.

Step 4 Adjust the transformation coefficient *R* and observe the output state of the above array system. As long as one of the phase trajectories jumps from the chaotic state to large-scale periodic state, it means that the value of the transformation coefficient at this time is the frequency value of the characteristic signal.

Step 5 After determining the frequency value of the characteristic signal, the array Duffing system for parameter estimation is designed according to the initial phase angle of the driving signal. At this time, the initial phase angles of the system drive signal are 0, π/2 and π, respectively. The frequency of the characteristic signal is reconstructed according to the transformation coefficient, that is, the frequency value is scaled on the time axis, so it does not affect the initial phase angle.

Step 6 Input the characteristic signal with noise after the previous step into the array Duffing system for parameter estimation, as shown in Equation (15), and observe the phase trajectory of output state of the Duffing system. The amplitude of the driving signal of the array Duffing system is trimmed to determine the corresponding driving signal amplitude while each Duffing equation jumps from the chaotic state to the large-scale periodic state, and is marked as *λ*_1_, *λ*_2_ and *λ*_3_ in turn.

Step 7 Finally, *λ*_1_, *λ*_2_ and *λ*_3_ is substituted into Equation (17) to obtain the amplitude and phase of the MWD signal.

## 4. Performance Evaluation and Discussion

The MWD signal detection method based on the improved array Duffing systems was verified by laboratory conditions and field measurement data in this section. Compared with original measurement data, the FIR filter and standard Duffing systems were conducted to evaluate the performance of the proposed improved array Duffing systems comprehensively.

### 4.1. Laboratory Testing

Under the laboratory conditions, the hardware-in-the-loop simulation (HILS) experimental platform is used to simulate attitude measurement signal in noise under the strong vibration environment in the pit, so as to comprehensively evaluate the performance of the chaotic effect based on array Duffing system in frequency detection and parameter estimation for weak signal detection. Furthermore, the inclination calculation is used for the sensor signal after chaos detection. The feasibility and effectiveness of the proposed method are proved by comparing the calculation results with the set values.

**Figure 4:**
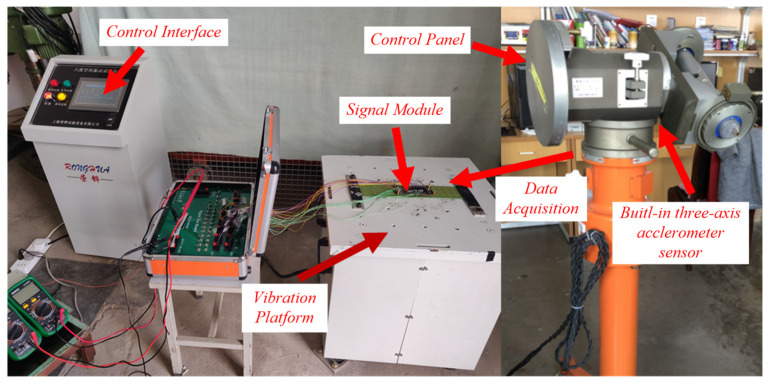
The main instrument and equipment in HILS.

The main equipment of the HILS experimental platform are shown in Figure 4, which includes a clinometer calibration, a six-dimensional space vibration experimental platform and its control device. The model of the clinometer is TX-3S, which is equipped with a three-axis accelerometer sensor, and the sensor parameters are shown in Table 1. By changing the angle of the control panel, the attitude of the drilling tool during the drilling process can be simulated, including inclination, azimuth and tool face angle. On the other hand, the space vibration platform could be set through the control interface to generate noise interference signals of different intensities, frequencies and gradient to simulate the strong vibration environment in the pit.

#### 4.1.1. Testing for Frequency Detection

(a) Testing for feasibility

By setting the control panel of the clinometer calibration, the real-time attitude angle of the drilling tool is simulated, and the output signal of the built-in three-axis accelerometer sensor is recorded by the storage oscilloscope. The X-axis accelerometer signal is selected as an example to carry out the simulation experiment in this section. It is assumed that the output signal of the X-axis at a certain time is expressed as *a*(*t*) = 0.02cos(10*t* − 2π/3). On the other hand, a random interference signal *n*(*t*) with a variance of 0.04 is generated by the six-dimensional space vibration experimental platform, whose variation range of frequency is from 0 to 10 Hz. It includes the part that is overlapped with the frequency of the characteristic signal. Therefore, the input signal of the Duffing system is obtained by the superposition of the X-axis accelerometer signal and noise signal as follows.

*I*(*t*) *=* 0.02cos(10*t* − 2π/3) + *n*(*t*) (18)

**Figure 5:**
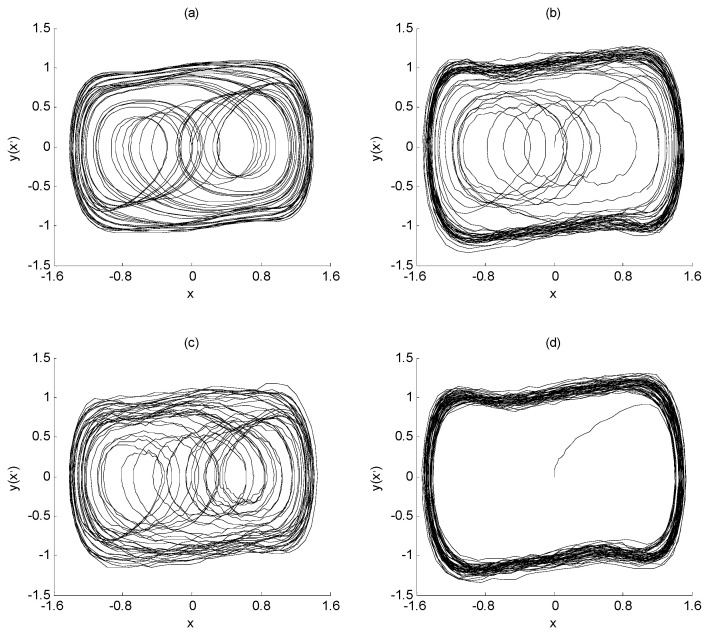
The output state while the characteristic signal takes different initial phase angle: (**a**) without characteristic signal and *β* = 0°; (**b**) including characteristic signal, *R* = 1 and *β* = −120°; (**c**) including characteristic signal, *R* = 10 and *β* = 0°; (**d**) including characteristic signal, *R* = 10 and *β* = −120°.

According to the calculation, the SNR of input signal *I*(t) is about −23 dB, which is consistent with the SNR condition of dynamic MWD. At this time, the characteristic signal is completely annihilated in the noise signal, and it is difficult to identify the frequency and other parameter of the characteristic signal by spectrum analysis. Therefore, the proposed variable scale array Duffing system is used to detect the frequency value of the characteristic signal based on its chaotic effect. The frequency detection model based on the array Duffing system is built using MATLAB/Simulink R2014a version, and the characteristic signal and noise signal are used as the model input. The initial parameters and simulation parameters of the model are shown in Table 2, the process of frequency detection is described in Steps 1 to 4 in Section 3. The frequency detection results are shown in Figure 5.

According to the simulation results, when the input signal *I*(*t*) is not considered and the initial phase angle of the drive signal is set to 0, the output result of the array Duffing system is chaotic, as shown in Figure 5a. At this time, the output state of the Duffing system does not change, and the system is in a critical state. After the input signal *I*(*t*), the scale transformation coefficient *R* is introduced at the same time, and the calculation step and sampling frequency are changed according to *R*. The array Duffing system is simulated and verified with the adjusted simulation parameters, and the output state of the system is observed when the initial phase angle of the drive signal is set to 0, 120° and −120°. The partial simulation results are shown in Figure 5b–d.

It can be seen from Figure 5b that only the initial phase angle of the driving signal is changed without the variable scale processing of the characteristic signal, and the output result of the Duffing system is still chaotic. It can be seen from Figure 5c that if the initial phase angle of the driving signal is incorrect, the system output result is still chaotic even if the characteristic signal is scaled. It can be seen from Figure 5d that when the scale transformation coefficient is set to 10 and the initial phase angle of the driving signal is set to −120°, the output state of Duffing system jumps to a large-scale period. It shows that there is a characteristic signal with an angular frequency of 10 rad/s in the input signal, which proves that the frequency detection method proposed in this paper is effective.

Further analysis of the detection results can lead to the following conclusions.

(1) Since the angular frequency of the characteristic signal is different from the driving signal, the scaling method is used to reduce the angular frequency of the characteristic signal to 1 rad/s, which can effectively solve the problem of large frequency parameters of the MWD signal. Moreover, when the output state of the Duffing system is the large scale period, the corresponding value of scale transformation coefficient is the angular frequency of the characteristic signal.
(2) On the premise that the scale transformation coefficient is correct, for the characteristic signal with amplitude greater than 0.02, the output state of a Duffing equation must be changed. For this example, the output state of the Duffing equation with an initial phase angle of −120° changes after the input signal. These results are consistent with the theoretical analysis.
(3) It can be seen from the simulation results that the high-intensity noise signal does not affect the output state of the array Duffing system, but only makes the phase trajectory of the Duffing system more rough.
(b) Testing for SNR threshold

In order to further explore the SNR threshold that the proposed method can handle when detecting frequency, the intensity of the noise signal is reset to increase its variance to 0.05, and all other parameters are the same as the previous section. At this time, the SNR of the input signal *I*(*t*) is about −24 dB.

According to the detection results in the previous section, the scale transformation coefficient and the initial phase angle of the array Duffing system are directly set to 10 and −120°, respectively.

**Figure 6:**
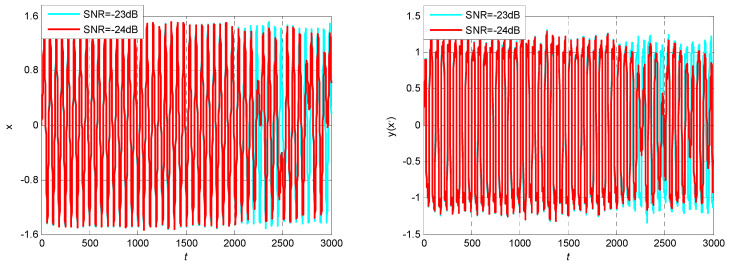
The time domain diagram of Duffing system output response with different SNRs.

Then, the characteristic signal with different SNRs is input into the frequency detection model for simulation, and the change trend of the Duffing system output state with time is shown in Figure 6. It can be seen that the simulation time is adjusted from 300 s to 3000 s due to the effect of scale transformation.

It can be seen from the simulation results that when the SNR is −23 dB, the system output response x changes periodically with time, ranging from about −1.6 to 1.6. In other words, the trend of change has obvious regularity. The other output response *y*(*x*’) is similar to *x*, which changes periodically with time, ranging from −1.3 to 1.3. If the two output responses *x* and *y* are plotted on a two-dimensional graph, it is obviously in the so-called large-scale periodic state.

When the SNR drops to −24 dB, the system output *x* still changes periodically with time in the first 2200 s, and the change trend is basically the same as that at −23 dB. However, the change trend showed partial disorder after 2200 s, with a range far less than ±1.6. The change trend of the other output *y* is similar, and disorder occurs after 2200 s. At this point, if the output response *x* and *y* are plotted on a two-dimensional graph, it is in the so-called chaotic state.

Therefore, when the amplitude of the characteristic signal is set as 0.02, the SNR threshold that the proposed method can identify its frequency is about −23 dB. In addition, through a large number of experimental verification, the following conclusions can be drawn: the lower the amplitude of the characteristic signal, the lower the SNR threshold that the proposed method can recognize.

#### 4.1.2. Testing for Parameter Evaluation

**Figure 7:**
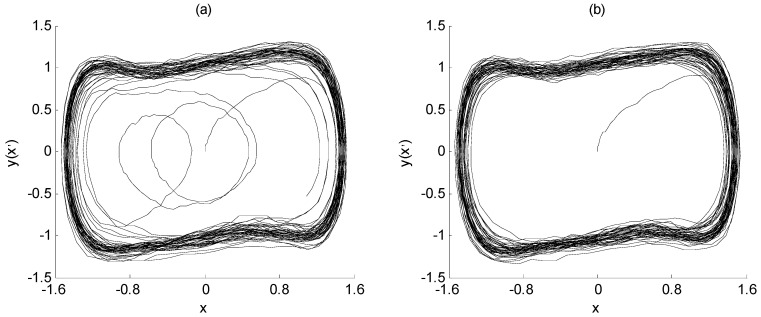
The output state of array Duffing equation I (*β* = 0°, *λ*_1_ = 0.7414): (**a**) SNR = −23 dB; (**b**) SNR = −21 dB.

**Figure 8:**
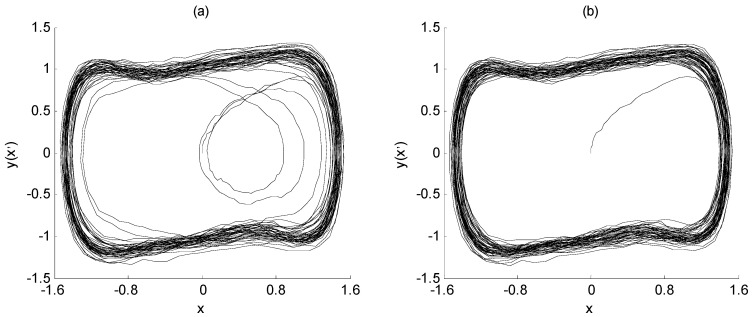
The output state of array Duffing equation II (*β* = 180°, *λ*_2_ = 0.7183): (**a**) SNR = −23 dB; (**b**) SNR = −21 dB.

**Figure 9:**
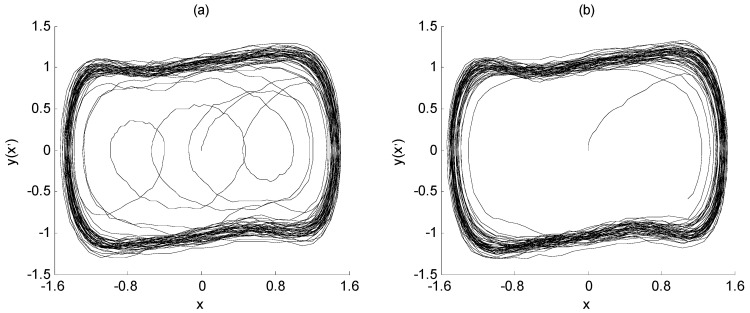
The output state of array Duffing equation III (*β* = 90°, *λ*_3_ = 0.7447): (**a**) SNR = −23 dB; (**b**) SNR = −21 dB.

When the frequency value of the characteristic signal is determined, the signal *I*(t) is still used as the input variables to input the array Duffing system for parameter estimation. The amplitude and phase value of the characteristic signal are estimated by the change in the system output state. The initial parameters and simulation parameters of the model are the same as those in the previous section. The implementation process of parameter estimation is described in Steps 5 to 7 in Section 3. The output state of the array Duffing system during parameter estimation is shown in Figure 7, Figure 8 and Figure 9.

During the simulation experiment, the signal *I*(*t*) is input into the three equations of the array Duffing system, and the critical values *λ*_1_, *λ*_2_ and *λ*_3_ of the driving signal amplitude are determined by observing the output state of the Duffing system.

First of all, the signal *I*(*t*) is input into array Duffing equation I, and the SNR of the characteristic signal is the same as that of the previous section, which is still −23 dB. When its driving signal amplitude is adjusted to 0.7414, the system output state is shown in Figure 7a. It is observed that the output state of the Duffing system is between the chaos and large-scale period. Compared with the chaotic state, the graph has an obvious periodization trend, and only has irregular changes in the final stage of simulation; so, it can be regarded as being in an approximate large-scale periodic state.

However, the approximate large-scale periodic state shown in Figure 7a is not enough to illustrate that 0.7414 is the critical value *λ*_1_ to be solved. Therefore, in order to determine the critical value, the intensity of noise signal *n*(*t*) is changed in the simulation experiment. By reducing the intensity of the noise signal, the SNR of the characteristic signal is increased from −23 dB to −21 dB. With other parameters unchanged, the value 0.7414 is still set as the critical value. At this time, the output state of the Duffing system is shown in Figure 8b. It can be seen from the phase trajectory that when the noise intensity decreases, the output state of array Duffing equation I shows an obvious large-scale periodic state, which proves that the critical value of the Duffing equation I is 0.7414.

The determination process of the critical value of Duffing equation II and Duffing equation III is the same as that of Duffing equation I. When the critical value *λ*_2_ is set to 0.7183 and the critical value *λ*_3_ is set to 0.7447, the output state of array Duffing equation II and III is between chaos and large-scale period, with an obvious periodization trend, as shown in Figure 8a and Figure 9a. When the SNR of the characteristic signal is improved, the output state of array Duffing equation II and III is in obvious large-scale periodic state, as shown in Figure 8b and Figure 9b. It is proved that the critical value *λ*_2_ is 0.7183 and the critical value *λ*_3_ is 0.7447.

**Figure 10:**
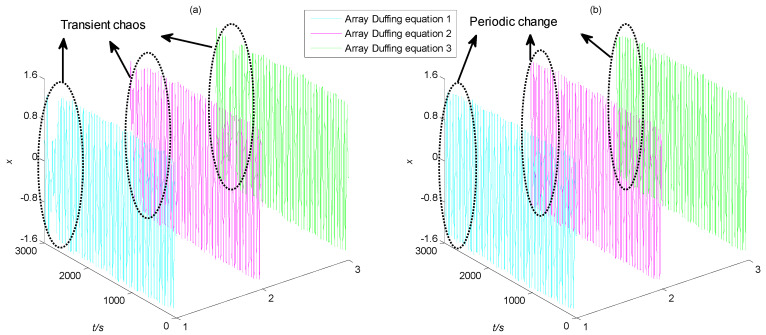
The time domain diagram of output term *x* of array Duffing system: (**a**) SNR = −23 dB; (**b**) SNR = −21 dB.

**Figure 11:**
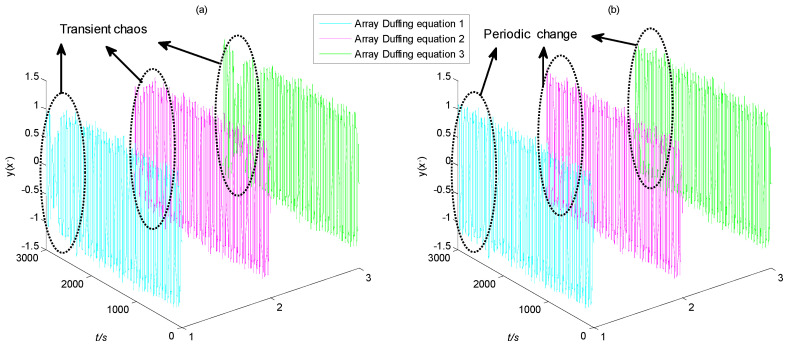
The time domain diagram of output term *y* of array Duffing system: (**a**) SNR = −23 dB; (**b**) SNR = −21 dB.

In order to further discuss the influence of noise intensity on the output state of the array Duffing system, the change curves of output x and y of the array Duffing equation in the time domain are shown in Figure 10 and Figure 11, respectively.

It can be seen from Figure 10a that when the SNR of the characteristic signal is −23 dB, the output term *x* of the three array Duffing equations changes periodically with time in the first 2700 s of the simulation time, with a range of −1.6 to 1.6. However, after about 2700 s, the trend of change is transient chaos, and the range of change is far less than ±1.6. On the other hand, it can be seen from Figure 10b that when the SNR of the characteristic signal is increased to −21 dB, the output term x of the three array Duffing equations change periodically with time during the whole simulation process, with a range of no more than ±1.6. The change trend has obvious regularity.

The curve of another output item *y* with time under different SNR conditions is shown in Figure 11. It can be seen that the change trend is very similar to output item *x*, but the change range is different. Therefore, the influence of noise on parameter estimation based on the chaotic effect is greater than that of frequency detection. Specifically, the SNR threshold of the detectable signal during frequency detection is −23 dB, which is lower than the SNR threshold of the detectable signal during parameter estimation, which is −21 dB.

According to the frequency detection model based on the array Duffing system, the amplitude of the driving signal is set to 0.72, and the adjustment accuracy is only 10^−2^. In this situation, the depth of the chaos state in the system is deeper. On the contrary, when the driving signal jumps to 0.73, the stability of the large-scale periodic state of the system is higher. In other words, the amplitude of the driving signal is relatively far from the critical amplitude of the system, and the system has strong anti-noise ability.

However, for the array Duffing system with parameter estimation, when the output state changes, the solved driving signal amplitude is 0.7414, 0.7183 and 0.7447, respectively, and the adjustment accuracy is 10^−4^. At this time, the degree of chaos or large-scale periodic state of the system is shallow. In other words, the amplitude of the driving signal is closer to the critical value, and then the anti-noise ability of the system is poor. Therefore, for the same noise intensity, irregular and disordered changes occur at the end of the simulation time. This is the main reason why the SNR threshold of parameter estimation is higher than that of frequency detection.

Therefore, considering the frequency detection and parameter estimation comprehensively, the SNR detection threshold based on the chaotic effect of the array Duffing system is −21 dB for the characteristic signal *a*(*t*).

Based on the analysis above, when the output state of the array Duffing system changes, the amplitudes of the three driving signals are *λ*_1_ = 0.7414, *λ*_2_ = 0.7183 and *λ*_3_ = 0.7447, respectively. By substituting *λ*_1_ and *λ*_2_ into Equation (17), the estimated value of the amplitude of the characteristic signal is 0.0188, and the first solutions of the initial phase angle are about ±128°. Then, by substituting *λ*_3_ into Equation (17), the second solutions of the initial phase angle of the characteristic signal are about −52° or −128°. By combining the solutions of the two sets of initial phase angles, the final estimated result of the initial phase angle is −128°.

Further statistics show the detailed error results for the parameters estimation, as shown in Table 3.

It can be seen from the statistical results in Table 3 that the amplitude and phase estimated based on the array Duffing system have high accuracy. The relative error of the amplitude of the characteristic signal is about 6%, and the relative error of the initial phase angle is about 6.7%.

#### 4.1.3. Testing and Comparison Analysis for Attitude Solution

In this section, the sensor signal after chaos detection is solved for the attitude of the drill bit, and the result is compared with that of the original measurement signal, FIR filter and standard Duffing system to evaluate the performance of the proposed method comprehensively.

First of all, the attitude parameters of the drilling tool while working vertically are simulated by the clinometer calibration, and the inclination is set to 5°. Then, the output signal of the three-axis accelerometer sensor is recorded by the storage oscilloscope and superimposed with the noise signal generated by the six-dimensional space vibration experimental platform. Thus, the attitude measurement signal under the background of strong noise is obtained. Finally, according to the chaos detection method described in Section 4.1 and Section 4.2, the frequency detection and parameter estimation of the three-axis accelerometer signal at 400 times are carried out, and the inclination of each data point is solved.

According to the experimental results in Section 4.1.1 and Section 4.1.2, the amplitude of the three-axis accelerometer signal used for attitude solution in this section is less than 0.02. Moreover, the SNR of the characteristic signal at the first 200 data points is set to −10 dB and the SNR at the last 200 data points is set to −20 dB, to verify the outstanding superiority of the chaos-detection method proposed in this paper, which is immune to noise signals.

**Figure 12:**
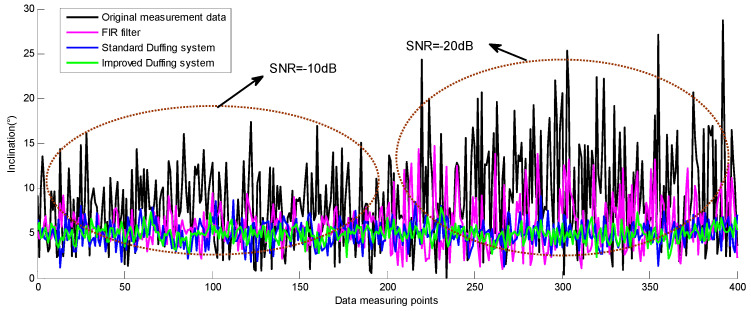
The solution results of the inclination under different algorithms.

**Figure 13:**
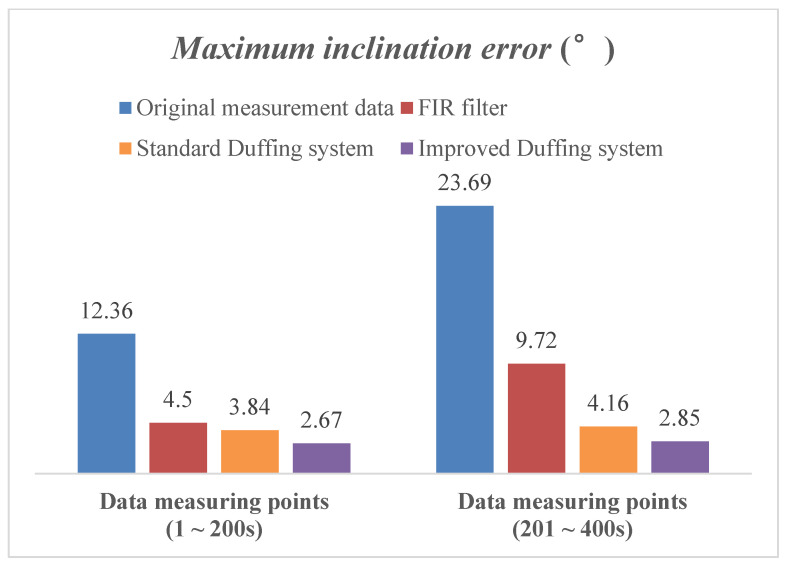
The statistical results of maximum inclination error by different algorithms.

**Figure 14:**
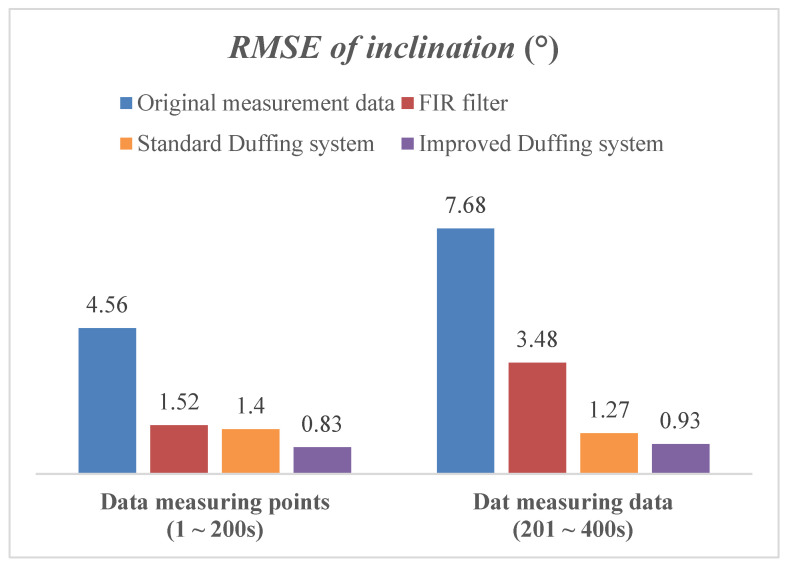
The RMSE of inclination by different algorithms.

The solution results of the inclination are shown in Figure 12. Further statistics show the detailed error results for the inclination, which are the maximum and root mean square error (RMSE) of the solution by these four algorithms, as shown in Figure 13 and Figure 14. When the SNR of the characteristic signal is −10 dB, it can be seen that the inclination fluctuates greatly and obviously deviates from the reference value while the original measured signal is directly calculated for attitude. At this time, the maximum error exceeds 10°. When the SNR of the characteristic signal is further reduced to −20 dB, the fluctuation amplitude of the inclination is further increased, and the maximum error is close to 24°. When using the FIR filter to process MWD signals, the strength of the noise signal has a significant impact on the calculation accuracy. When the SNR of the signal to be measured is −10 dB, the maximum error of the inclination is 4.5. However, when the SNR further drops to −20 dB, this value diverges sharply to 9.72.

But on the contrary, the Duffing detection system is used to process the MWD signal, and the maximum inclination error is almost the same regardless of whether the SNR of the measured signal is −10 dB or −20 dB. The solution results are very close to the reference value during the whole simulation process. It can be seen that the change in noise intensity has no obvious influence on the solution result of inclination. This is mainly because the array Duffing system is not affected by noise intensity while detecting weak signal. According to its detection principle, increasing the noise intensity will make the output track of Duffing system rough, but will not affect the accuracy of frequency detection and parameter estimation. The simulation result indicates that the Duffing system indeed has good immunity to noise. In addition, the improved Duffing system proposed in this article results in smaller calculation errors, and both the maximum error and RMSE are better than the solution results of the standard Duffing system.

The above simulation results and analysis demonstrate that the weak signal detection method based on the chaotic effect of the array Duffing system is indeed immune to noise signals and sensitive to weak characteristic signals. As long as the SNR does not exceed the detectable threshold of chaos detection, the proposed method can effectively improve the accuracy of the inclination solution.

### 4.2. Field-Drilling Testing

**Figure 15:**
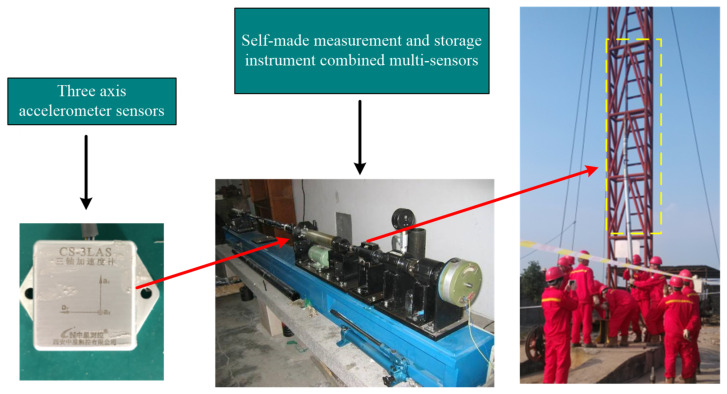
Schematic of the field-drilling test.

In order to further verify the performance of the proposed weak signal detection method for dynamic MWD signal, field-drilling data were used for testing and analysis. The experimental data came from the field-drilling process of a well in northern Shaanxi. The field-acquisition process and installation position of the triaxial accelerometer sensors are shown in Figure 15, and the drilling environment parameters are listed in Table 4. During drilling, the guiding tool was in a stable and straight state.

The CS-3LAS sensor, which was developed by the Zhongxing measurement and control company, was chosen as the accelerometer in this test. The accelerometer is suitable for the specific requirements of downhole drilling. The specific parameters of the sensors are shown in Table 5.

**Figure 16:**
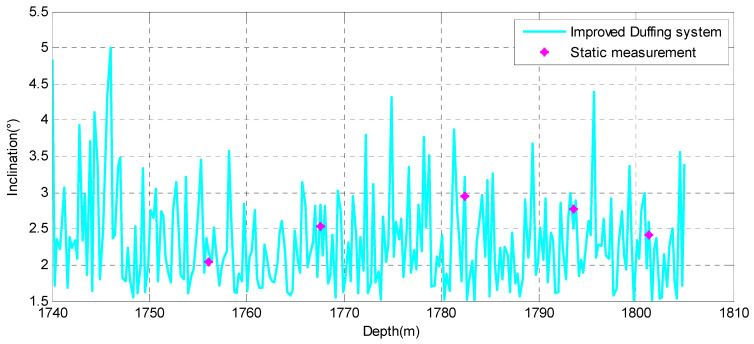
The solution results of inclination by the improved Duffing system.

In order to further evaluate the overall performance of the proposed method, the data after the improved Duffing system was implemented were used to obtain the real-time inclination of the drilling tool. There was no interference of vibration acceleration in the attitude measurement of the stopping of the drilling, which ensures the accuracy of the attitude parameters, such as inclination. Therefore, it was used as a reference value to verify the detective performance of the improved Duffing system. The solution results of the inclination obtained by the proposed algorithm are given in Figure 16. As a comparison, the static measurement results of five data points are also given in Figure 16. As a comparison, the results of other algorithms for these five points were also statistically analyzed, including FIR filtering and the standard Duffing system. The statistical results are shown in Table 6.

From the statistical results in Table 6, it can be seen that the solution results processed by the FIR filter have a significant error, with a relative error of 14.23–30.32%, indicating that this method is indeed greatly affected by vibration noise. The solution accuracy processed by the standard Duffing system is slightly higher than that of FIR filtering, but due to its poor sensitivity to sinusoidal signals, the relative error is mostly between 10–20%. However, the improved Duffing system proposed in this article obtains the best solution results, with relative errors of basically less than 10% for all five points. This shows that the proposed method in this paper provides a new solution to the detection problem of weak SNR signals during MWD.

## 5. Conclusions

The contributions of this paper are as follows.

(1) An improved array Duffing system is developed by combining the technique of scale transformation to solve the problem that the frequency value of the MWD signal is too large, and it also make the detection window cover all phases to solve the influence of the initial phase angle of the MWD signal on frequency detection. Experimental results indicate that the proposed method can effectively detect the frequency value of the MWD signal in strong noise interference.
(2) Three equations with different initial phases of the driving signal are combined to form another array Duffing system, which synchronously estimates the amplitude and phase of the MWD signal, and then recovers the complete MWD signal. Experimental results demonstrate that the proposed estimation method has high accuracy and the SNR can be as low as −21 dB when the amplitude of the MWD signal is greater than or equal to 0.02.
(3) Simulation and field-drilling test results and comparison analysis validate that the proposed methodology can effectively curb the adverse impacts of multi-frequency and high-intensity vibration noise for weak signal detection, leading to higher solution accuracy for the inclination with dynamic MWD.

## Figures and Tables

**Table 1:** Sensor parameters.

Parameters	Values
Range	±20 g
Scale factor	50 ± 5 mv/g
Non-linearity	≤0.5% Fs
Calibration	≤0.01 g
Bandwidth	≤1 kHz
Zero-bias (25 °C)	1.5 ± 0.1 V
Zero-bias temperature drift	±1 mg/°C
Startup time	≤1 s
Range	±20 g

**Table 2:** Simulation parameters.

Categories	Parameters	Values
Duffing chaos model	Damping *k*	0.5
	Nonlinear coefficient *a*	1
	Nonlinear coefficient *b*	1
Drive signals of Duffing system	Critical amplitude *λ_c_*	0.72
	Angular frequency *ω*	1 rad/s
	Initial phase angle	*β* = 0, ±120°
Simulation preliminary	Initial value of system output	*x*(0) = 0; *x’*(0) = 0
	algorithm	Ode4 (Runge-Kutta)
	Calculation step *T*	0.1 s
	Sampling frequency *f*_s_	10 Hz
	Simulation time	300 s

**Table 3:** The error statistical results of parameters estimation.

Characteristic Signal	*λ*_1_	*λ*_2_	*λ*_3_	Amplitude		Initial Phase Angle		SNR Threshold
				Estimated Result	Relative Error	Estimated Result	Relative Error	
*a*(*t*)	0.7414	0.7183	0.7447	0.0188	6%	−128°	6.7%	−21 dB

**Table 4:** Basic parameters of the field-drilling test.

Parameters	Value
Well depth	1740–1805 m
WOB	10 MPa
Downhole temperature	40 °C
Pump pressure	6.6 MPa
Drilling fluid density	1.15 g/cm^3^
Suspended load	79 kN
Operation time	75 h
Rotary speed	120 rpm
The setting value of inclination	2.5°

**Table 5:** Characteristics of the accelerometer sensors.

**The Performance Index**	Axial	X	Y	Z
	Range	±3 g (±1~±100 g)		
	Bandwidth	0 to ≥500 Hz		
	Scale factor	300 ± 30 mV/g		
	Calibration	≤1 mg		
	Non-linearity	≤0.3% Fs		
	Zero-bias (25 °C)	1.5 ± 0.1 V		
	Zero-bias temperature drift	±1 mg/°C		
	Startup time	≤0.001 s		
**Environmental Characteristics**	Work temperature	−40 °C~+70 °C		
	Storage temperature	−40 °C~+125 °C		
	Anti-crash (0.5 ms)	10^4^ g		
**Physical Characteristics**	Weight	40 g		
	Size	19.5 × 18 × 10 mm		

**Table 6:** Error statistics of the inclination in field-drilling tests.

Algorithm	Attitude Parameter	Depth/m				
		1756.12	1767.56	1782.38	1793.56	1801.36
Static Measurement	Inclination (°)	2.04	2.53	2.95	2.77	2.41
FIR filter	Inclination (°)	2.50	2.17	2.41	1.93	3.12
	Relative error	22.55%	14.23%	16.61%	30.32%	29.46%
Standard Duffing system	Inclination (°)	2.32	2.09	3.23	2.15	2.01
	Relative error	13.73%	17.39%	9.49%	22.38%	16.60%
Improved Duffing system	Inclination (°)	2.18	2.83	3.21	2.50	2.59
	Relative error	6.86%	11.86%	8.81%	9.75%	7.47%

## Data Availability

Some or all data, models, or code generated or used during the study are proprietary or confidential in nature and may only be provided with restrictions.
